# Enhanced fullerene–Au(111) coupling in (2√3 × 2√3)R30° superstructures with intermolecular interactions

**DOI:** 10.3762/bjnano.6.147

**Published:** 2015-06-29

**Authors:** Michael Paßens, Rainer Waser, Silvia Karthäuser

**Affiliations:** 1 Peter Grünberg Institut (PGI-7) and JARA-FIT, Forschungszentrum Jülich GmbH, 52428 Jülich, Germany; 2 IWE 2 and JARA-FIT, RWTH Aachen University, Sommerfeldstraße 24, 52056 Aachen, Germany

**Keywords:** adatom–vacancy mechanism, differential conductance, fullerene, Ising model, scanning tunnelling microscopy

## Abstract

Disordered and uniform (2√3 × 2√3)R30° superstructures of fullerenes on the Au(111) surface have been studied using scanning tunneling microscopy and spectroscopy. It is shown that the deposition and growth process of a fullerene monolayer on the Au(111) surface determine the resulting superstructure. The supply of thermal energy is of importance for the activation of a Au vacancy forming process and thus, one criterion for the selection of the respective superstructure. However, here it is depicted that a vacancy–adatom pair can be formed even at room temperature. This latter process results in C_60_ molecules that appear slightly more bright in scanning tunnelling microscopy images and are identified in disordered (2√3 x 2√3)R30° superstructures based on a detailed structure analysis. In addition, these slightly more bright C_60_ molecules form uniform (2√3 x 2√3)R30° superstructures, which exhibit intermolecular interactions, likely mediated by Au adatoms. Thus, vacancy–adatom pairs forming at room temperature directly affect the resulting C_60_ superstructure. Differential conductivity spectra reveal a lifting of the degeneracy of the LUMO and LUMO+1 orbitals in the uniform (2√3 x 2√3)R30° superstructure and in addition, hybrid fullerene–Au(111) surface states suggest partly covalent interactions.

## Introduction

Monolayers of close-packed fullerenes on metal surfaces belong to one of the most extensively studied self-assembled systems due to their rich structural and electronic properties [1]. A considerable interest in C_60_ films arises from their use in photovoltaic cells [2–3] and potential applications in molecular electronics [4]. Likewise, C_60_ molecules can be used as chemical anchoring groups to bind functional molecules to electrodes and thus, to construct electronic circuits. In this case, charge transport takes place through the fullerenes and crucially depends on the electrode coupling of C_60_ [5–7]. Therefore, it is essential to understand in detail the interactions at the C_60_–metal interface.

First systematic studies of close packed fullerene thin films on Au(111) surfaces using scanning tunnelling microscopy (STM) were performed by Altman and Colton [8–10]. They observed two structural arrangements, the (2√3 × 2√3)R30° and the uniform (7 × 7)R0° superlattices, with the unit cell of the C_60_ overlayer aligned along the [11−2] and [10−1] directions of the Au(111) surface, respectively. Low energy electron diffraction (LEED) measurements by Tzeng et al. [11] revealed a R14° structure, which was confirmed by STM measurements later on [12–13]. In addition, the structure could be identified as a (√589 × √589)R14.5° lattice [12] with 49 molecules.

Usually, the periodicity and orientation of molecular self-assembled superstructures with respect to the underlying substrate are identified by the apparent height of the respective molecules using STM technique [14]. However, in case of fullerenes it is also possible to identify the orbital symmetry, whose appearance strongly depends on the rotational orientation of single C_60_, like shown in simulations of empty-state STM-images of free fullerenes [15]. Therefore, it was possible to identify this (√589 × √589)R14.5° superlattice formed by fullerenes adopting 11 different orientations and it could be shown that intermolecular interactions play a major role in stabilizing this structure [12].

Another interesting fact is that fullerenes with two different apparent heights in STM images, usually referred as “bright” and “dim” C_60_ [8,16], are observed in different structural domains. In a systematic study Gardener et al. [16] proposed, that the reason for the dim C_60_ are nanopits, also called vacancies, which are formed at the C_60_–Au interface. Dim C_60_ molecules adsorbed at vacancies are lower in height than the bright C_60_ molecules and, even more interesting, a pronounced bias dependence of the apparent height difference between both is observed [16], which points to an increased charge transfer at the interface between dim C_60_ and Au. The creation of vacancy structures by adsorption of C_60_ is also observed on other metal surfaces, such as Ag(111) [17–18], Cu(111) [19–20] and Pt(111) [21–23], and therefore seems to be the rule rather than the exception. Moreover, it could be shown by STM investigations that the self-assembly of C_60_ on Au(111) surfaces causes the partial or complete lifting of the herringbone reconstruction [16] or the forming of a new surface arrangement at the C_60_–Au interface depending on the thermal treatment [24–25].

The number and the distribution of dim C_60_ molecules adsorbed on vacancies differ in the respective superstructures on Au(111). While very few dim C_60_ molecules are randomly scattered within bright C_60_ molecules in the (7 × 7)R0°-superstructure, they show a “quasi-periodic” distribution in the (√589 × √589)R14.5° structure [12–13]. In contrast, the (2√3 × 2√3)R30° superstructure reveals a large number of dim C_60_ with randomly scattered bright C_60_ and, therefore, this structure is commonly called “disordered”. Interestingly, this (2√3 × 2√3)R30° superstructure shows a dynamic bright–dim flipping near room temperature, which points to highly mobile vacancies at the C_60_–Au(111) interface [16,26]. Recently, also a uniform (2√3 × 2√3)R30° structure with all C_60_ molecules exhibiting the same contrast was observed [16,26]. This uniform R30° structure is recognized to have a small offset of about 1° with respect to the disordered R30° structure.

The hexagonal Au(111) surface structure and the mobility of Au vacancies in combination with the inherent rotational degrees of freedom of C_60_ give rise to a large variety of possible C_60_–Au(111) superstructures. Remarkably, the rotational orientations of C_60_ are not random but depend sensitively on the interface and intermolecular interactions. In order to gain more insights into the formation and stability of possible superstructures of C_60_ on Au(111) surfaces, we investigate in detail the orbital symmetry and thus, the rotational orientation and interface interactions of fullerenes in disordered and uniform (2√3 × 2√3)R30° superstructures. These structural investigations are complemented by conductance measurements sheding new light on the fullerene–Au(111) interface.

## Results and Discussion

### (2√3 × 2√3)R30° superstructures

**Figure 1:**
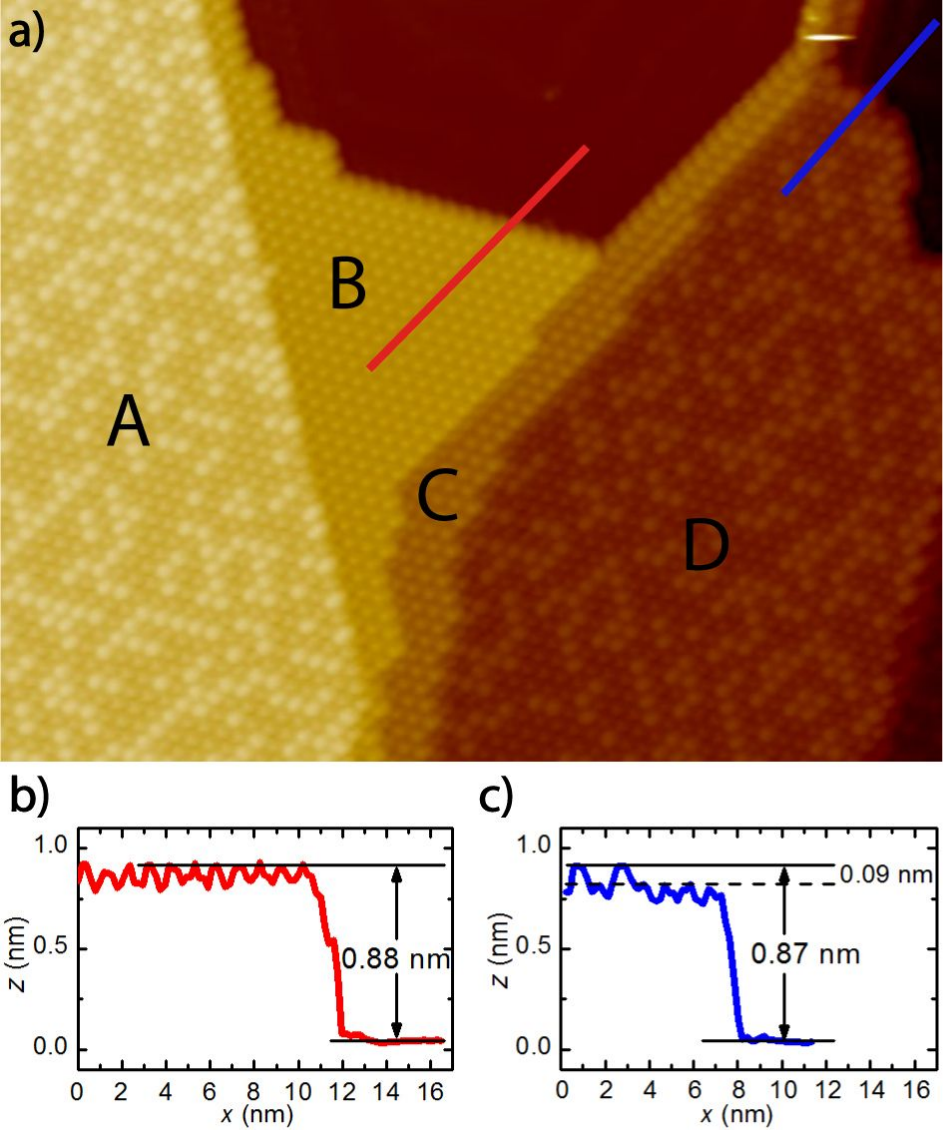
(a) (54.5 × 40.5 nm^2^) STM image of uniform (marked by B and C) and disordered R30° domains (A and D) (*U*_bias_ = 1.1 V, *I*_T_ = 0.18 nA; sample A2). Linescans measured along the indicated red (b) and blue line (c) in Figure 1a reveal that the apparent height differences between a uniform R30° domain and the Au(111) surface or between a disordered R30° domain and the Au(111) surface, respectively, are comparable at *U*_bias_ = 1.1 V. The apparent height difference between bright and dim C_60_ in disordered R30° domains corresponds to approximately 0.09 nm.

High-resolution STM offers the opportunity to study in detail the structure of molecular monolayers, including domains and their boundaries. In Figure 1 an overview STM image of a C_60_ monolayer assembled on an Au(111) single crystal surface according to the procedure given in the experimental part is shown (sample A2). Using these experimental conditions the formation of the less stable (7 × 7)R0° and (√589 × √589)R14.5° superstructures is effectively suppressed. In the large-scale image only (2√3 × 2√3)R30° superstructures (hereafter referred to as R30°) can be identified, two disordered and two different uniform ones, which are marked by capital letters. While the disordered R30° structures (A and D in Figure 1) cover large Au(111) terraces, only small domains of the uniform analogues, B and C, are found in corners of Au(111) terraces or on domain boundaries of disordered R30° structures, respectively.

First, we concentrate on the disordered R30° superstructure with clearly distinguishable dim and bright C_60_ molecules, which is considered in literature to be the most stable structure. The apparent height difference between dim and bright C_60_ molecules is obtained from cross-sectional profiles measured along the indicated directions in Figure 1. It amounts to 0.09 nm at a bias voltage *U*_bias_ = 1.1 V and is in full agreement with values given in literature [16,26]. Accordingly, the “dim” C_60_ are assigned to molecules adsorbed with the hexagon parallel to the Au(111) surface on a single-atom vacancy (hex-vac), while the “bright” C_60_ are assumed to be adsorbed with the 6:6 C–C bond on top positions of the unreconstructed Au(111) surface (6:6-top) [26], as it is largely accepted in literature. The apparent height difference between hex-vac and 6:6-top C_60_ of about 0.1 nm, as it was measured for *U*_bias_ > 1 V, can be explained as follows: The height difference due to different molecular orientations, i.e., between hex-top and 6:6-top, amounts to 0.04 nm caused by the orientation-dependent radii of C_60_ [26–27]. In addition, there is a considerable height difference, which depends on the adsorption site, of 0.05 nm between top and vacancy positions on the Au(111) surface. Thus, an apparent height difference around 0.1 nm is expected based on the given surface structure and is indeed measured for a positive sample bias (*U*_bias_ > 1.0 V), when electrons are tunneling from the tip into the lowest unoccupied molecular orbital (LUMO) of C_60_.

However, the apparent height difference between hex-vac and 6:6-top C_60_ depends strongly on the applied bias voltage and exhibits the steepest slope around *U*_bias_ = 0.5 V. This behavior is attributed to different electronic structures of bright and dim molecules [16,28] though it could not be confirmed by DFT calculations [29–30], which indicate only a minor charge transfer from the substrate to the molecule. The origin of the voltage-dependent apparent height differences determined by STM and the origin for the existence of dim and bright molecules in the most stable R30° superstructure is still under debate. In the following, we will develop a model based on our experimental observations supplemented by data from literature, that will help to understand most features observed so far in commensurate (2√3 × 2√3)R30° superstructures of C_60_ on Au(111) surfaces and that may be adopted also to other metal surfaces.

### Ising model

**Figure 2:**
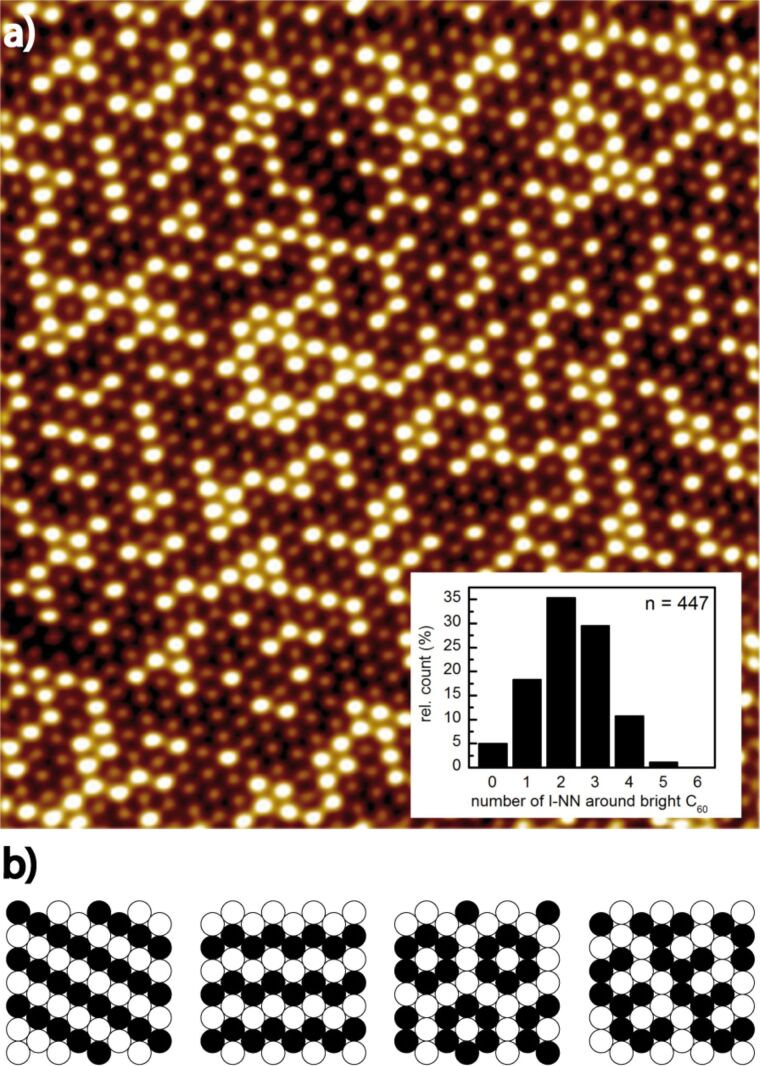
(a) Filtered STM image (30 × 35 nm^2^) to highlight the dim and bright contrast of the C_60_ molecules in a disordered R30° domain (*U*_bias_ = 0.68 V, *I*_T_ = 0.11 nA ; sample A2). Inset: histogram of the number of like next neighbors (l-NN) around bright C_60_ molecules. (b) Characteristic structure elements observed in the disordered ground state of frustrated Ising systems: ideal line, kinked line, pinwheel and comb structure.

One point of discussion is the existence of 6:6-top C_60_ molecules in the disordered R30° superstructure despite the fact that this position is obviously less stable than the hex-vac position. Here, advantageous entropy effects [26] or an adlayer buckling in order to minimize the lattice mismatch to the underlying substrate are given as possible explanations. However, solely entropy effects should result in completely random structures while a lattice mismatch of only a few percent causes usually highly ordered superstructures, both unlike the surface structure shown in Figure 2. Here, special features, such as kinked lines of bright C_60_ molecules, comb structures, or ring like structures of bright C_60_ molecules with a dim C_60_ molecule in the centre (pinwheel) can be observed. Most interestingly, this distribution of dim and bright C_60_, like often observed for disordered R30° structures, is typical for the disordered ground state of frustrated Ising systems on a triangular lattice [31–33].

A two-dimensional Ising model can be used to describe the arrangement of any units that are able to interact with the nearest neighbors (NN), can adopt two different states, and are arranged on a regular surface. Due to the fact that in Ising systems on a hexagonal grid not all nearest neighbor interactions can be minimized at the same time, these systems are frustrated. This is for example observed for antiferromagnetic systems on triangular lattices [33], i.e., systems with preferred interactions between unlike NN. Due to lattice constrictions the energy can only be minimized locally and the ground state of the system is manifold degenerated. In our case the units are the C_60_ molecules and the two different states are “bright” and “dim”. The NN energy is small, but not negligible. According to [26] the adsorption energy of C_60_ in the 6:6-top position amounts to 0.99 eV and to 1.26 eV in the hex-vac position, but to 1.17 eV in the case of a 6:6-top C_60_ with a hex-vac neighbor (assuming that the adatom resulting from the vacancy formation is located on the surface). This points to a small adsorption energy gain for 6:6-top C_60_, if they are surrounded by unlike NN, i.e., hex-vac C_60_. On the other hand, a pure domain consisting only of C_60_ molecules in a hex-vac position would be most favorable referring to the internal energy (*U*). However, such a pure hex-vac C_60_ domain on Au(111) has not been observed so far, and a possible reason might be the vacancy formation energy of 0.63 eV to 1.11 eV [26,30], which has to be provided first. Furthermore, entropy (*S*) effects might play a role during the C_60_ monolayer preparation process at elevated temperatures (*T*), so that a pure hex-vac C_60_ domain on Au(111) might not correspond to the minimum free energy (*F* = *U* − *TS*) under these conditions. Nevertheless, 6:6-top C_60_, which prefer to be surrounded by unlike NN (unl-NN), and hex-vac C_60_, which prefer to be surrounded by like next neighbors (l-NN), form surface structures that remind of Ising-like systems (Figure 2). This is even more astonishing, since in the standard Ising model either l-NN (interaction energy, *J* > 0) or unl-NN interactions (*J* < 0) are preferred for both states, i.e., bright and dim in our case. Obviously, fullerenes on Au(111) belong to a more complicated Ising-like system.

In order to verify the visual impression of the STM images we performed a statistical analysis of the distribution of bright and dim C_60_ shown in Figure 2. C_60_ molecules form a triangular surface lattice with six NN on Au(111)-substrates. In the case of a ratio *x* = 0.5 and *J* < 0 (for bright C_60_) the number of l-NN in various ground state configurations of frustrated Ising systems would be l-NN_Ising_ = 2 (see Figure 2b). This number has to be compared to a value of l-NN_random_ = 3 in the case of a random distribution of l-NN. In our case, the ratio of 6:6-top C_60_ amounts to *x* = 0.42 and the number of l-NN_Ising_ for the bright C_60_ molecules, 2.25 ± 0.05, is derived from Figure 2a. This value has to be compared to a possible random distribution characterized by l-NN_random_ = 2.52, which would be expected without NN interactions or at high temperatures. The obtained number of l-NN_Ising_ from Figure 2a shows a small, but statistically significant deviation from a random distribution and points to the fact, that the disordered (2√3 × 2√3)R30° superstructure of C_60_ on Au(111) can be described by an Ising-like model. This indicates that in the discussed superstructure small NN interactions exist and that the system does not adopt the lowest possible energy in the disordered (2√3 × 2√3)R30° superstructure.

### Influence of the deposition procedure on the C_60_ superstructure formation

Starting from the usually observed disordered R30° superstructure a lower energy state could be reached if the ratio of dim C_60_ molecules in a hex-vac position could be increased and thus, finally a pure domain of dim C_60_ molecules would result. This suggestion is supported by the superstructures of dim C_60_ molecules observed on Pt(111) and Cu(111) surfaces after an annealing step at 1100 K [22] and 560 K [34], respectively. Moreover, a successive change from bright to dim C_60_-molecules on the Cu(111)-surface was monitored during annealing cycles and attributed to a reconstruction [32].

However, a domain of dim C_60_ molecules in hex-vac positions on the Au(111) surface can only be assembled, if enough vacancies are available. In order to create the required vacancies elevated temperatures can advantageously be applied during the C_60_ monolayer preparation process, as shown in [11] or [16]. Both could demonstrate, that the disordered R30° superstructure with an increased amount of dim fullerenes, compared to the uniform (7 × 7)R0° or the quasi-periodic (√589 × √589)R14.5° superstructure (hereafter referred to as R0° and R14.5°, respectively), is favored after an annealing step at higher temperatures (Table 1). Without a post-annealing step the R0° domain is dominant, but after an annealing step to 570 K the yield of R14.5° and dis-R30° domains increases and after annealing to 680 K the dis-R30° domain is overwhelmingly dominant [16].

**Table 1:** Comparison of key preparation parameter and resulting C_60_-superstructures.

reference	*T*_substrate_/K	C_60_ flux /ML·min^−1^	*T*_annealing_/K	obtained superstructure^a,b^	ratio of bright C_60_ in dis-R30°

[11]	rt	0.05	none	R0°, R14.5°	
			470	R0°, R14.5°, dis-R30°	
			620	R0°, R14.5°, dis-R30°	
			690	**dis-R30°**	

[16]	rt		none	R0°, R14.5°, dis-R30°, u-R30°	0.49^c^
			570	R0°, R14.5°, dis-R30°, u-R30°	0.33^c^
			680 (1–2 h)	R14.5°, **dis-R30°**	0.35^c^

[35]	rt	0.2	540	R0°, R14.5°, dis-R30°	0.25

[26]	rt	1	none	R0°, R14.5°, dis-R30°, u-R30°	
	670	3.6	none	dis-R30°	0.40
	640		660	**dis-R30°**	0.20

this work (A)	440	0.1	none	**dis-R30°**, u-R30°	0.43
this work (A2)			480 (80 min)	**dis-R30°**, u-R30°	0.42
this work (A3)			rt (≥5 days)	**dis-R30°**, u-R30°	0.25
this work (B)	rt	0.25	rt (≥5 days)	R0°, R14.5°, dis-R30°, u-R30°	0.26

^a^The C_60_-superstructures are abbreviated as follows: R0° = uniform (7 × 7)R0° superstructure, 4 molecules per unit cell, a few dim C_60_ scattered around R14.5° = quasi-periodic (√589 × √589)R14.5° superstructure, 49 molecules per unit cell, ratio of bright C_60_, *x* = 0.86 [16] dis-R30° = disordered (2√3 × 2√3)R30° superstructure, one molecule per unit cell, ratio of bright C_60_ = variable, energetically favourable [16,26] u-R30° = uniform (2√3 × 2√3)R30° superstructure, one molecule per unit cell, ratio of enhanced-bright (en-bright) C_60_, *x*_en_ = 1. ^b^The favoured superstructure is underlined, the all-dominant superstructure is given in bold face. ^c^These values are deduced from Figure 1 in [16].

Following this argumentation it should be also possible to increase the ratio of dim C_60_ in the dis-R30° superstructure with increased deposition temperature or a subsequent annealing step. Therefore, we listed the key preparation conditions and the structural parameters of our dis-R30° superstructure in comparison with literature data in Table 1. In the last column in Table 1 the ratio of bright C_60_ in the dis-R30° superstructure is given and indeed, a rough correlation between this ratio and the temperature treatment of the sample can be deduced. It is possible to decrease the amount of bright C_60_ by deposition at elevated temperatures and/or a post-annealing step. However, the finally determined ratio of bright C_60_ in the dis-R30° structure varies between 0.2 and 0.35 and does not correlate to the annealing temperature. Here, we assume that the cooling process, which is not mentioned in most references, is also of relevance. If fullerenes forming a dis-R30° superstructure can be described as an Ising-like system, as mentioned in the previous section, the ratio of bright C_60_ will be increased at elevated temperatures due to entropy effects. This process counteracts the forming of dim C_60_ based on the increased availability of vacancies at these temperatures. As a consequence, the lowest possible ratio of bright C_60_ may be 0.35 for temperatures around 670 K, and 0.20 for rt. In the case of a rapid cooling of the C_60_ sample the ratio of bright molecules achieved at elevated temperatures is preserved while it may change otherwise.

A decrease of the amount of bright C_60_ corresponds to an increase of the amount of dim C_60_ and thus, the lowering of the system energy. Most interestingly, we could observe that the dis-R30° superstructure does not only transfer into a lower energy state after a high temperature treatment, that is after a thermally activated vacancy forming process, but also after storage at room temperature for several days. At room temperature the energy for vacancy formation on clean substrates is not supplied. However, at room temperature the switching of C_60_ from bright–to-dim and vice versa, involving the diffusion of a vacancy, was observed [16,26] and we suggest that this mechanism is also important for the forming of a lower energy dis-R30° structure.

**Figure 3:**
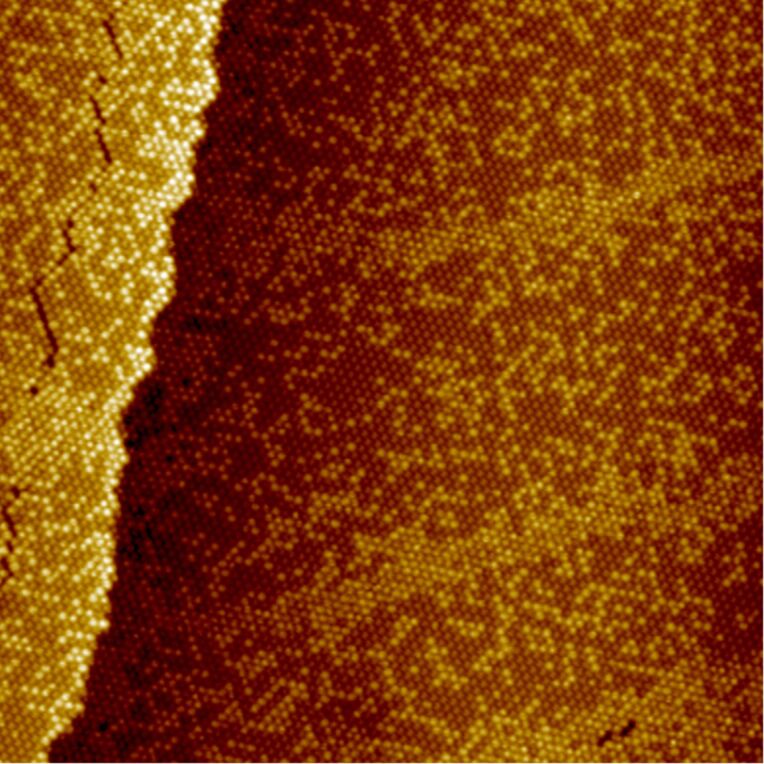
STM image (76 × 76 nm^2^) of a large dis-R30° domain with a low ratio of bright molecules and stripes consisting of accumulated bright molecules (*U*_bias_ = 1.96 V, *I*_T_ = 0.10 nA, sample A3). Furthermore a crack is visible within the dis-R30° domain.

UHV-STM images taken after a thermal treatment of the C_60_ monolayer on the Au(111) surface and an additional conditioning at rt (Figure 3, sample A3) revealed very large domains of the dis-R30° superstructure (150 × 150 nm^2^). Moreover, these domains can be classified into two regions, a dis-R30° superstructure with a rather low ratio of bright C_60_ and additionally stripes of accumulated bright C_60_, which appear in quasi-periodic distances of about 30 nm. Besides this, also cracks in the C_60_ monolayer were observed, which presumably were formed during the cooling down to 77 K, due to higher expansion coefficients of C_60_ monolayers compared to Au(111) [36–37].

**Figure 4:**
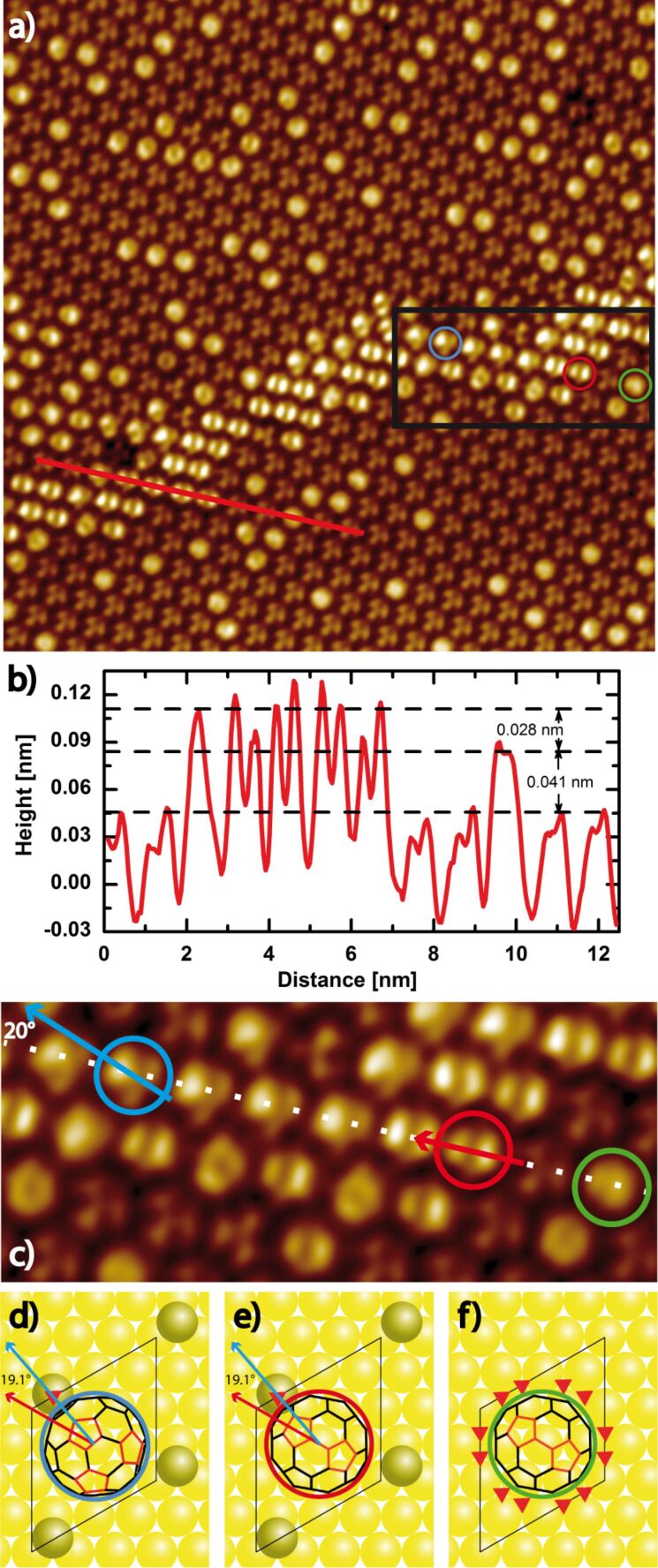
(a) High resolution STM Image (24 × 24 nm^2^) of a stripe structure (*U*_bias_ = 0.63 V, *I*_T_ = 0.07 nA, slightly low pass filtered, sample A3). (b) The linescan shows the apparent height difference (0.028 nm) between en-bright C_60_ molecules within the stripe and bright C_60_ molecules within the dis-R30° structure at *U*_bias_ = 0.63 V. (c) Enlarged STM image of the C_60_-stripe structure marked by a black rectangle in Figure 4a. (d–f) Top view models of selected C_60_ molecules on the Au(111) surface marked by colored circles in Figure 4a and Figure 4c. (d) C_60_ in 5:6-top position (encircled in blue) with a mirror plane rotated by about 20° referenced to the red C_60_. (e) One three-fold hollow site is occupied by an adatom next to the 6:6-top C_60_ (encircled in red). (f) 6:6-top C_60_ characteristic for the dis-R30° structure, encircled in green. All available three-fold hollow sites around this C_60_ are indicated by red triangles.

A closer inspection of a characteristic region of sample A3 with stripes after 5 days at room temperature (Figure 4) reveals that the dis-R30° superstructure, especially in the vicinity of the stripes, gets poor in bright C_60_ (*x* = 0.25). This depletion in combination with the periodic assembly of the stripes points to a thermodynamic activated process, like the diffusion of vacancies. However, a simple diffusion of vacancies cannot explain that the ratio of bright C_60_ in the combined area of dis-R30° superstructure and stripe amounts to only *x* = 0.32 instead of 0.42, like observed before (see Figure 2a, sample A2). Obviously, not only a separation between bright and dim C_60_ took place, but additionally, a vacancy forming process has to be considered, which involves the formation of a vacancy–adatom pair at room temperature. The linescan taken across the stripe structure, like indicated in Figure 4, gives further insights. The bright C_60_ forming the stripe structure appear 0.028 nm higher than those of the dis-R30° superstructure, i.e., the 6:6-top C_60_ (at *U*_bias_ = 0.63V). These enhanced bright (en-bright) C_60_ observed in the stripe structures have not been described so far. Like the difference in apparent heights between dim and bright C_60_ also the apparent height difference between bright and en-bright C_60_ is bias dependent pointing to differences in molecular energy levels.

The en-bright C_60_ appear by far less bright than the “extra bright” C_60_ observed on Au(111) terraces [38] or “superbright” C_60_ molecules on Ag adatom islands described in [39]. The apparent height differences between these extra bright or superbright C_60_ and bright molecules amount to 0.24 nm or to 0.12 nm (0.19 nm) for negative (positive) bias, respectively. Since no adatom islands are identified here, we presume that an en-bright C_60_ is formed by the interaction of an Au adatom with a C_60_-molecule on the Au(111) surface resulting in an energetically preferred adsorption geometry. The energy gained by the conversion of two 6:6-top C_60_ into one hex-vac and one en-bright C_60_ seems to compensate the energy that is necessary for the forming of a vacancy–adatom pair. This hypothesis is supported by the fact that the number of the en-bright C_60_ in Figure 4a corresponds exactly to the number of additional vacancies formed during the conditioning of sample A3 at room temperature for 5 days, like discussed before.

Interestingly, the en-bright C_60_ adsorb often with the 6:6 C–C bond on top positions of the Au(111) surface (6:6-top). However, there seems to be a subtle equilibrium between different adsorption geometries with almost comparable energy that also allows for the adsorption of C_60_ with the 5:6 C–C bond on the Au(111) surface. In Figure 4c one row of en-bright C_60_ is highlighted, which shows a continuous change from a slightly tilted 6:6-top (red circle) to a 5:6-adsorption position (blue circle). Based on this change in adsorption positions we developed a model to explain the observations. In Figure 4f a schematic of the Au(111) surface with a C_60_ molecule adsorbed in a 6:6-top position is shown (green circle). A new Au adatom will occupy one of the three-fold hollow sites (fcc or hcp position) on the Au(111) surface, like shown in Figure 4f. An occupancy of one of the three-fold hollow sites with an Au adatom is shown in Figure 4e. Here, the interaction of the C_60_ with the adatom is favored. However, if the C_60_ is tilted by only about a few degrees around the C5-axis the adsorption position assumed in Figure 4d is reached, i.e., the C_60_ is adsorbed with the 5:6 C–C bond on a top-position of the Au(111)-surface (5:6 top). The appearance of the 5:6-top C_60_ in STM corresponds to the C_60_ marked with a blue circle in Figure 4d.

This scenario is promoted by a first-principles study of C_60_ on a Pt(111) surface [40]. Here, it was concluded that Pt adatoms resulting from vacancy–adatom formation are located in the interstitial regions between the C_60_ molecules on the Pt(111) surface. Thus, additional Pt–C covalent bonds form between C_60_ and Pt adatoms and further stabilize the reconstructed surface. In addition, new DFT calculations of C_60_ on Au(111) reveal [30] that the missing energy to thermodynamically allow for the vacancy–adatom formation is only 0.29 eV. This energy might be available, if the process described above is regarded, that is, two 6:6-top C_60_ are transformed to one hex-vac C_60_ and one 5:6-top (or 6:6-top) C_60_ attached to an adatom. In conclusion, we assume that the adsorption of C_60_ with the 5:6 bond or a 6:6 bond on the Au(111) surface with adjacent Au adatoms is energetically favorable. Furthermore, the accumulation of en-bright C_60_-molecules in the stripe structure points to a favored l-NN interaction for en-bright C_60_ molecules in contrary to bright C_60_ molecules.

### Uniform R30° domain

**Figure 5:**
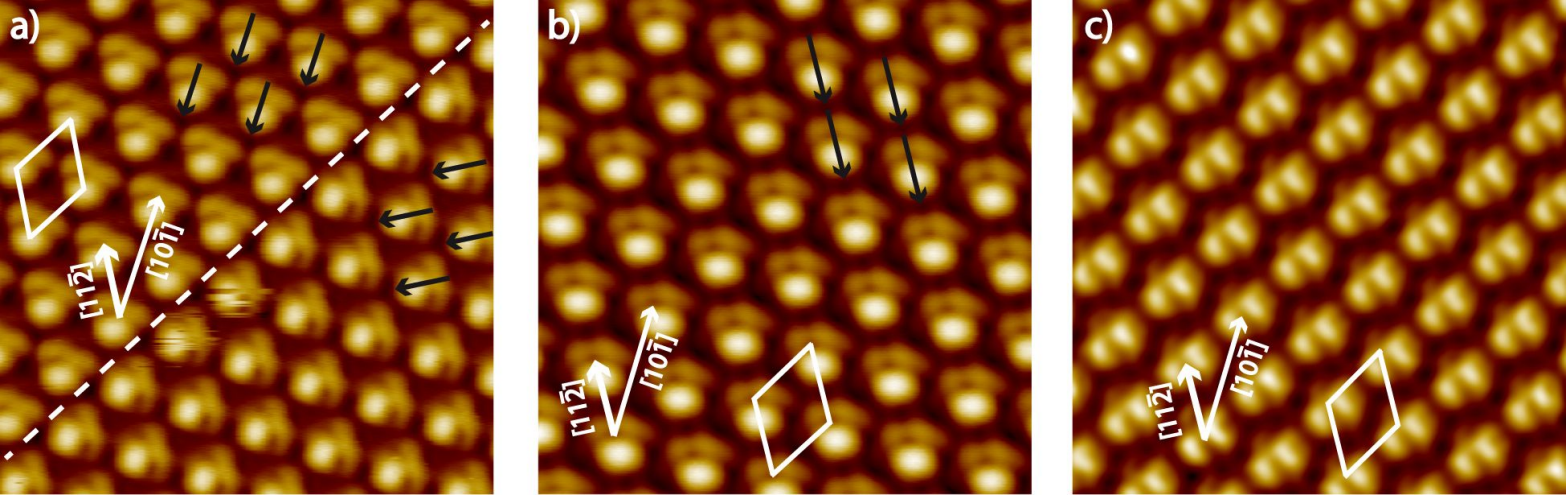
(a) High resolution STM image (7 × 7 nm^2^) of two u-R30° domains of C_60_ adsorbed with the 5:6 C–C bond on Au(111) (*U*_bias_ = 0.46 V, *I*_T_ = 1.0 nA, sample B), which are rotated by 60° to each other. The arrows indicate the orientation of the molecules. (b) STM image (6.25 × 6.25 nm^2^) of a second u-R30° structure (*U*_bias_ = 0.33 V, *I*_T_ = 0.08 nA, sample A2), which is formed by a 30° rotation of the C_60_ with respect to the C_60_ in Figure 5a. c) (6.8 × 6.8 nm^2^) STM image of a third u-R30° structure formed by C_60_ adsorbed with the 6:6 C–C bond and slightly tilted (*U*_bias_ = 0.80 V, *I*_T_ = 0.41 nA, sample B). All structures point to an intermolecular effect that explains the long range order in the u-R30° superstructure.

In sample A only the very large dis-R30° and two different u-R30° superstructures were present. The latter were observed predominantly in the corners of gold terraces and at gold edges next to dis-R30° structures as shown in Figure 1a. Each u-R30° superstructure presents only one orbital symmetry. In Figure 5 high resolution UHV-STM images of u-R30° superstructures of C_60_ adsorbed with the 5:6 or the 6:6 bond on the Au(111) surface are shown. The left image reveals two domains, which are rotated by an angle of 60° implicating commensurability with the Au(111) surface. The domain boundary is only defined by the orbital geometry of the C_60_ with respect to the surface but no positional defect at the domain boundary is observed. Even though the two different domains are stable, the domain boundary fluctuates. The C_60_ molecules at the domain boundary can flip between the two orbital orientations back and forth, presumably due to the influence of the tip while scanning the surface. This points to a small potential barrier for the flipping. The C_60_ molecules building the uniform domain exhibit the same orbital symmetry like the molecules marked by the blue circle in Figure 4c. Therefore, we assume that the u-R30° superstructure in Figure 5a is formed by C_60_ adsorbed with the 5:6 bond in combination with Au adatoms. Most interestingly, a symmetry breaking is observed in this u-R30° structure due to the adsorption of C_60_ molecules with the 5:6 C–C bond (with one mirror plane as symmetry element) on a surface position with a C3-axis as symmetry element.

With respect to the C_60_ shown in Figure 5a, whose mirror plane is parallel to the [10−1] axis of the Au(111) surface the C_60_ in Figure 5b are rotated by 30° (mirror plane parallel to the [11−2] axis of Au(111)). C_60_ in Figure 5c show a slightly tilted two-lobe structure, resulting from C_60_ in 6:6 positions. The appearance of the C_60_ forming this domain has striking similarities with the molecule marked with a red circle in Figure 4c.

### Spectroscopy

**Figure 6:**
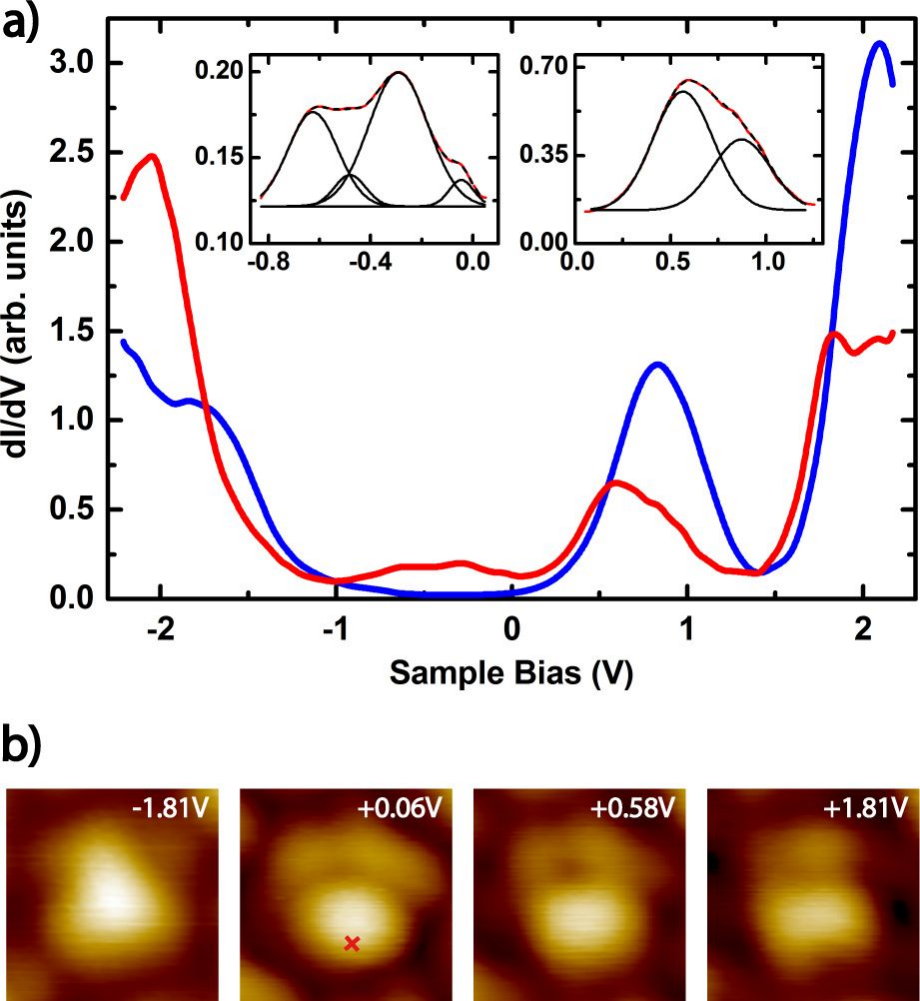
(a) Differential conductance spectra (*dI*/*dV*) over a C_60_ molecule as indicated by the red cross in Figure 6b (red curve: *U*_bias_ = 0.45 V, *I*_T_ = 0.16 nA, 20 mV, 724 Hz; blue curve: *U*_bias_ = 0.46 V, *I*_T_ = 0.20 nA, 50 mV, 213 Hz). Inset: Gauss-curves fitted at the LUMO and the interface states. (b) C_60_ orbital structure within the u-R30° domain at different *U*_bias_ as indicated in the picture (*I*_T_ = 0.06–0.17 nA) (1.05 × 1.05 nm^2^).

The molecular orbitals of fullerenes are highly degenerated in the gas phase due to the high molecular symmetry. The HOMOs and the LUMOs exhibit a degeneracy of 5 and 3, respectively, which should be lifted, if intermolecular interactions or molecule–surface interactions become relevant. However, as reported so far, the electronic configuration of C_60_ remains largely unperturbed upon adsorption and the charge transfer to C_60_ on Au(111) is small, which also has been confirmed by DFT calculations [29–30]. For reference purposes we first probed the *dI*/*dV* spectra of a bright C_60_ molecule. A typical spectrum showing molecular resonances of bright C_60_ is displayed in Figure 6 (blue). It is in full agreement with *dI*/*dV* spectra reported in literature for bright C_60_ embedded in islands [28,41–42]. The obtained orbital energies for the HOMO, the LUMO, and the LUMO+1 amount to −1.83 eV, 0.84 eV, and 2.08 eV, respectively. The LUMO exhibits a typical peak shape of 0.56 full width half maximum (FWHM).

In addition we performed STS measurements on the u-R30° superstructures composed of en-bright C_60_, like depicted in Figure 5. The resulting *dI*/*dV*-curve is also shown in Figure 6 (red). Obviously the HOMO and LUMO energies are shifted with respect to bright C_60_ to lower energies and are located at −2.06 eV, 0.60 eV, and 1.8 eV. This shift of the molecular energy levels is in great accordance with the increased brightness of the en-bright C_60_, that is, the difference in apparent height with respect to the bright 6:6-top C_60_, as shown in Figure 4b. The HOMO–LUMO gap is almost unaffected by the given superstructure environment and remains at 2.7 eV, as well as the FWHM of the LUMO does not change significantly. However, the LUMO and LUMO+1 peak shapes of the red curve show a clear structure, as depicted in Figure 6. We used Gauss curves to fit these peaks and found resonances at 0.57 eV and 0.87 eV as well as at 1.80 eV and 2.10 eV. This points to the lifting of the degeneracy of the LUMOs, even though the charge transfer from Au(111) to the LUMO of C_60_ is not assumed to be largely increased since the LUMO peak is not broadened. In fact, the lifting of the degeneracy of the LUMO t_1u_ orbitals is likely caused by the breaking of the symmetry in the special adsorption position of the en-bright C_60_ with a nearby Au adatom, as discussed above for the u-R30° domain (Figure 5).

In the case of the adsorption of a single C_60_ in an adatom geometry, DFT based calculations [43] indicate also a splitting of the LUMO orbitals and a shift to lower energies while the HOMO–LUMO gap is not affected. These calculations predict a weaker partial charge transfer for C_60_ in contact with flat Au(111) surfaces than for adatom geometries, which is in accordance with our findings. However, our assumed geometry with an adatom on an Au(111) surface in contact with a C_60_ is located in between the two scenarios assumed in literature [43], i.e., a flat Au(111) surface and an adatom contact.

Another feature of the *dI*/*dV*-spectrum of C_60_ in the u-R30° domain (Figure 6, inset) has not been discussed so far. There is a relatively broad peak with low intensity just below *E*_F_, between 0.0 eV and −0.8 eV, which has not been observed so far for C_60_ molecules in other superstructures. This broad peak can be fitted by four Gauss curves centered at −0.63 eV, −0.48 eV, −0.29 eV, and −0.04 eV, and can be attributed to hybrid molecule–metal states, likely formed between occupied molecular orbitals and Au(111) surface states. In the considered energy range Shockley-type surface states are available on clean Au(111) surfaces (ca. 0.4 eV) [44–45]. The dispersion of the hybrid C_60_–Au(111) states is presumably caused by the non-equivalent interactions of the C_60_ molecules in the u-R30° domain with the Au adatom and the Au(111) surface, and due to the lifted symmetry. However, if a partly covalent interaction is established between the Au surface or the adatom and the C_60_, this could lead to an additional charge transfer and a realignment of the molecular orbitals (i.e., charge redistribution) with respect to the Fermi energy of the metal, like observed here. Furthermore, a newly created adatom–C_60_ unit may be polar and therefore, would give rise to intermolecular dipol–dipol interactions. These polar interactions in combination with the symmetry breaking are assumed to result in the formation of a polar surface structure, namely the u-R30° superstructure with a long-range order.

## Conclusion

In conclusion, we could show that the deposition and growth process of a fullerene monolayer on Au(111) is of great importance for the resulting interface geometries and thus, the self-assembly process which results in the formation of the respective superstructure. The dis-R30° superstructure is described by a two-dimensional Ising-like system of hex-vac and 6:6-top C_60_. The ratio of hex-vac C_60_ is a measure of the availability of the most favorable hex-vac adsorption sites, which may be limited by the use of a room temperature deposition and growth process followed by a rapid cooling of the C_60_ monolayer. During thermal treatment at elevated temperatures the formation of Au vacancies is enabled and an increase of the ratio of hex-vac C_60_ is observed, which corresponds to a lowering of the system energy. However, a vacancy formation process based on the creation of a vacancy–adatom pair is observed even at room temperature. We propose that this process is energetically feasible because of the energy gain resulting from the conversion of two 6:6-top C_60_ into one hex-vac and one en-bright C_60_ through interaction with the vacancy and the adatom, respectively. The en-bright C_60_ accumulate in the dis-R30° superstructure forming stripes and moreover, build domains which can be identified as u-R30° superstructures. These superstructures exhibit relevant intermolecular interactions, likely mediated by Au adatoms. The observed symmetry breaking causes the lifting of the degeneracy of the LUMO and LUMO+1 orbitals in the differential conductance spectra. In addition, hybrid fullerene–metal states are identified and attributed to partly covalent interactions between adatoms on the Au(111) surface and C_60_ molecules. We hope, that these investigations will cause theoretical studies, which may give a detailed analysis of the interfacial and intermolecular interactions discussed here.

## Experimental

Low-temperature scanning tunnelling microscopy (STM) and spectroscopy (STS) experiments were carried out with a commercial Createc STM (Germany) operated in ultra-high vacuum (UHV) with a base pressure of 1 × 10^−10^ mbar. All STM images were obtained in constant-current mode at 77 K sample temperature using a custom-made electrochemically etched tungsten tips. The *dI*/*dV* spectra were recorded through lock-in detection of the ac tunnelling current achieved by modulating the sample bias after switching off the feedback loop. The single crystal Au(111) substrate (MaTecK, Germany) was cleaned in UHV by cycles of Ne^+^ ion sputtering (1 kV, 10 min) and thermal annealing (600 °C, 20 min). The cleanliness was checked by STM inspection revealing a Au(111) surface with large terraces and the well-known (23 × √3) herringbone reconstruction.

C_60_ molecules (Sigma Aldrich, purity 99.9 %) were outgassed and then deposited by sublimation at 320 and 350 °C using a Knudsen cell, with the Au(111) surface heated to 170 °C for sample A and at room temperature for sample B. The C_60_ deposition rate was 0.1 ML/min for sample A and 0.25 ML/min for sample B, respectively, as monitored by a quartz crystal microbalance. During the deposition the background pressure was in the 10^−10^ mbar range. After deposition the samples were transferred into the LT-UHV-STM and measured at 77 K. After a first inspection, sample A was post-annealed at 210 °C for 80 min and characterized again (denoted as sample A2). Thereafter, it was stored at room temperature for at least five days and subsequently monitored (sample A3). Without previous high temperature treatment sample B was also stored at room temperature for at least five days.

## References

[1] Shi X-Q, Van Hove M A, Zhang R-Q (2012). J Mater Sci.

[2] Thompson B C, Fréchet J M J (2007). Angew Chem, Int Ed.

[3] Deibel C, Dyakonov V (2010). Rep Prog Phys.

[4] Salinas M, Halik M (2013). Appl Phys Lett.

[5] Martin C A, Ding D, Sørensen J K, Bjørnholm T, van Ruitenbeek J M, van der Zant H S J (2008). J Am Chem Soc.

[6] Bilan S, Zotti L A, Pauly F, Cuevas J C (2012). Phys Rev B.

[7] Leary E, La Rosa A, González M T, Rubio-Bollinger G, Agraït N, Martín N (2015). Chem Soc Rev.

[8] Altman E I, Colton R J (1992). Surf Sci.

[9] Altman E I, Colton R J (1993). Phys Rev B.

[10] Altman E I, Colton R J (1993). Surf Sci.

[11] Tzeng C-T, Lo W-S, Yuh J-Y, Chu R-Y, Tsuei K-D (2000). Phys Rev B.

[12] Schull G, Berndt R (2007). Phys Rev Lett.

[13] Zhang X, Yin F, Palmer R E, Guo Q (2008). Surf Sci.

[14] Lüssem B, Müller-Meskamp L, Karthäuser S, Waser R (2005). Langmuir.

[15] Wang H, Zeng C, Wang B, Hou J G, Li Q, Yang J (2001). Phys Rev B.

[16] Gardener J A, Briggs G A D, Castell M R (2009). Phys Rev B.

[17] Li H I, Pussi K, Hanna K J, Wang L-L, Johnson D D, Cheng H-P, Shin H, Curtarolo S, Moritz W, Smerdon J A (2009). Phys Rev Lett.

[18] Pussi K, Li H I, Shin H, Serkovic Loli L N, Shukla A K, Ledieu J, Fournée V, Wang L L, Su S Y, Marino K E (2012). Phys Rev B.

[19] Pai W W, Jeng H T, Cheng C-M, Lin C-H, Xiao X, Zhao A, Zhang X, Xu G, Shi X Q, Van Hove M A (2010). Phys Rev Lett.

[20] Xu G, Shi X-Q, Zhang R Q, Pai W W, Jeng H T, Van Hove M A (2012). Phys Rev B.

[21] Felici R, Pedio M, Borgatti F, Iannotta S, Capozi M, Ciullo G, Stierle A (2005). Nat Mater.

[22] Liu C, Qin Z, Chen J, Guo Q, Yu Y, Cao G (2011). J Chem Phys.

[23] Pinardi A L, Biddau G, Van De Ruit K, Otero-Irurueta G, Gardonio S, Lizzit S, Schennach R, Flipse C F J, López M F, Méndez J (2014). Nanotechnology.

[24] Gimzewski J K, Modesti S, Gerber C, Schlittler R R (1993). Chem Phys Lett.

[25] Torrelles X, Pedio M, Cepek C, Felici R (2012). Phys Rev B.

[26] Shin H, Schwarze A, Diehl R D, Pussi K, Colombier A, Gaudry É, Ledieu J, McGuirk G M, Serkovic Loli L N, Fournée V (2014). Phys Rev B.

[27] Adams G B, O’Keeffe M, Ruoff R S (1994). J Phys Chem.

[28] Schull G, Néel N, Becker M, Kröger J, Berndt R (2008). New J Phys.

[29] Lu X, Grobis M, Khoo K H, Louie S G, Crommie M F (2004). Phys Rev B.

[30] Kaiser A, Viñes F, Illas F, Ritter M, Hagelberg F, Probst M (2014). Nanoscale.

[31] Han Y, Shokef Y, Alsayed A M, Yunker P, Lubensky T C, Yodh A G (2008). Nature.

[32] Pai W W, Hsu C-L, Lin M C, Lin K C, Tang T B (2004). Phys Rev B.

[33] Ottaviano L, Teodoro C D, Santucci S, Profeta G (2003). Phys Low-Dimens Struct.

[34] Hashizume T, Motai K, Wang X D, Shinohara H, Saito Y, Maruyama Y, Ohno K, Kawazoe Y, Nishina Y, Pickering H W (1993). Phys Rev Lett.

[35] Tang L, Xie Y, Guo Q (2011). J Chem Phys.

[36] Kwon Y-K, Berber S, Tománek D (2004). Phys Rev Lett.

[37] Shin H, O'Donnell S E, Reinke P, Ferralis N, Schmidt A K, Li H I, Novaco A D, Bruch L W, Diehl R W (2010). Phys Rev B.

[38] Tang L, Xie Y, Guo Q (2012). J Chem Phys.

[39] Li H I, Abreu G J P, Shukla A K, Fournée V, Ledieu J, Serkovic Loli L N, Rauterkus S E, Snyder M V, Su S Y, Marino K E (2014). Phys Rev B.

[40] Huang M (2012). Phys Chem Chem Phys.

[41] Torrente I F, Franke K J, Pascual J I (2008). J Phys: Condens Matter.

[42] Franke K J, Pascual J I (2012). J Phys: Condens Matter.

[43] Géranton G, Seiler C, Bagrets A, Venkataraman L, Evers F (2013). J Chem Phys.

[44] Everson M P, Davis L C, Jaklevic R C, Weidian S (1991). J Vac Sci Technol, B.

[45] Chen W, Madhavan V, Jamneala T, Crommie M F (1998). Phys Rev Lett.

